# Humanized Rodent Models for Cancer Research

**DOI:** 10.3389/fonc.2020.01696

**Published:** 2020-09-11

**Authors:** Huimin Tian, Yanan Lyu, Yong-Guang Yang, Zheng Hu

**Affiliations:** ^1^Key Laboratory of Organ Regeneration & Transplantation of Ministry of Education, The First Hospital of Jilin University, Changchun, China; ^2^National-Local Joint Engineering Laboratory of Animal Models for Human Diseases, Changchun, China; ^3^International Center of Future Science, Jilin University, Changchun, China

**Keywords:** humanized mice, rodent, immunology, cancer, immunotherapy

## Abstract

As one of the most popular laboratory animal models, rodents have been playing crucial roles in mechanistic investigations of oncogenesis as well as anticancer drug or regimen discoveries. However, rodent tumors show different or no responses to therapies against human cancers, and thus, in recent years, increased attention has been given to mouse models with xenografted or spontaneous human cancer cells. By combining with the human immune system (HIS) mice, these models have become more sophisticated and robust, enabling *in vivo* exploration of human cancer immunology and immunotherapy. In this review, we summarize the pros and cons of these humanized mouse models, with a focus on their potential as an *in vivo* platform for human cancer research. We also discuss the strategies for further improving these models.

## Introduction

The high morbidity and mortality rates associated with various cancers across the globe clearly indicate that cancer-related research is one of the fastest-growing fields in the world (1). Although great progress has been made in understanding the underlying mechanisms of cancer and the discovery of anticancer drugs in the past few decades, efficient clinical translation of these technologies remains very limited (2). One of the main reasons for this is that most of these studies rely on rodent models, which have a number of important physiological differences from humans. Thus, rodent cancer models cannot accurately simulate the physiology of cancer patients, which necessitates the development of novel animal models that are better equipped to precisely and comprehensively represent the complex feature of human cancers allowing for improved basic and translational investigations (3).

Animals bearing tumors of human origin were first developed using T-cell-deficient nude mice inoculated with human cancer cell lines [now known as cell-derived xenograft (CDX) models] and these rodent models became a popular *in vivo* platform to study human oncogenesis and test anticancer drug efficacy (4). Moreover, development of mouse strains with more severe immunodeficiencies, such as NOD/SCID mice and NOD/SCID IL2rg^−/−^ mice, further facilitated the use of rodents to efficiently repopulate primary human cancer samples or cells and served to mirror the heterogenous features of these cancers in patients [patient-derived xenograft (PDX) models] (5). The application of CDX and PDX models in cancer research markedly facilitates human cancer biology investigations and anticancer drug interventions. However, more recent analysis has revealed that the absence of human immune elements in these models may severely compromise their value in translational research and the development of novel human cancer immunotherapies (6).

It is widely accepted that the immune system is closely related to oncogenesis and cancer prognosis as well as response to anticancer therapies (7). Key breakthroughs in cancer immunotherapies, including co-stimulatory molecule blockade (8) and chimeric antigen T (CAR-T) cell transfer (9), further highlight the importance of including human immunity in cancer research. Humanized mice are novel animal models designed to address some of these concerns, thereby making them an attractive alternative for biomedical research (10). Briefly, humanized mice are engineered to carry human genes, cells, or tissues, allowing them to directly mirror human physiological and pathological characteristics (10). Humanized mice with functional human immune systems (HIS mice) could be a powerful model for understanding the interaction between human immune components and human cancer and contributing to anticancer intervention development (11, 12).

Here, we have focused on cancer studies that use humanized mice with functional human immune reconstitution and discuss their advantages and disadvantages and prospect their advancement in the future.

## Humanized Mice With Human Cancer Development

### Immunodeficient Mice

Robust xenogeneic immune rejection is a major barrier to the engraftment of human cancer cells in immune-competent rodents (13). Several immunodeficient murine strains have been developed by disrupting the relevant genes crucial for immune cell development/survival/function. The ability to construct these immunodeficient animals is a cornerstone in producing humanized mice for evaluating human cancer development. The characteristics of these immunodeficient murine models have been reviewed in detail in earlier studies (10). Briefly, immunodeficient mice were designed to overcome the rejection of human cancer cells mediated by the mouse adaptive (T and B cells) and innate (NK cells and macrophages) immune responses (11). For example, elimination of the forkhead box N1 (*Foxn1*) gene (4), recombination activating gene 1 (*Rag1*) (14), recombination activating gene 2 (*Rag2*) (15), protein kinase, and DNA activated and catalytic polypeptide (*Prkdc*) genes (16) results in mice with T and/or B cell deficiency; deletion of interleukin 2 receptor subunit gamma (*IL2rg*) (17) or β2-microglobulin (*B2m*) (18) genes leads to the absence or functional impairment of mouse NK cells, whereas selection of non-obese diabetic (NOD) mouse background (19) or knock-in human (20) or NOD (21) *Sirpa* genes prevents phagocytosis by mouse macrophages. Combinations of these genetic engineering strategies have been applied to develop the popular immunodeficient mouse strains, such as NOD/*Prkdc*^*s*^^cid^ (NOD/SCID), NOD/SCID IL2rg^−/−^ (NSG or NOG), and Balb/c Rag1^−/−^ IL2rg^−/−^ (BRG) that have all been used in human oncology studies (11) (Table 1).

**Table 1:** Immunodeficient mouse strains for human cancer study.

**Name**	**Strain**	**T**	**B**	**NK**	**Macrophage (for human cells)**	**Complement**	**References**
Nude	Foxn1^null^	No	Yes	Yes	Phagocytose	Yes	(4)
Scid	B6.CB17-Prkdc^scid^/SzJ	No	No	Yes	Phagocytose	Yes	(22)
BRG	BALB/c.Rag2^−/−^ ${\text{IL-2Rg}}_{\text{c}}^{-/-}$	No	No	No	Partial tolerant	Yes	(11)
NOD-scid	NOD.CB17-Prkdc^scid^/J	No	No	Function impaired	Tolerant	No C5	(23)
NOD/SCID B2m^null^	NOD.Cg-B2m^tm1Unc^Prkdc^scid^/SzJ	No	No	Function loss	Tolerant	No C5	(18)
NSG	NOD.Cg-Prkdc^scid^IL2rg^tm1Wjl^/SzJ	No	No	No	Tolerant	No C5	(24)
NOG	NOD.Cg-Prkdc^scid^IL2rg^tm1Sug^/JicTac	No	No	No	Tolerant	No C5	(25)
BRGS	BALB/c.Rag2^−/−^${\text{IL-2Rg}}_{\text{c}}^{-/-}$NOD.sirpa	No	No	No	Tolerant	Yes	(21)
hSIRPa-BRG	BALB/c.Rag2^−/−^${\text{IL-2Rg}}_{\text{c}}^{-/-}$human.sirpa	No	No	No	Tolerant	Yes	(20)

### CDX and PDX Mouse Models

Based on the type of human cells or samples used in the transplantation, immunodeficient mice grafted with human cancer cells can be classified as CDX or PDX mouse models (4, 5). Following *in vitro* culture of human cancer cell lines for many passages, they can easily form human tumors in most T-cell deficient immunodeficient mouse strains, making them a valuable initial model for cancer investigation. For example, in order to study the underlying mechanisms why *BRAF* mutations are co-related with aggressive, less-differentiated, and therapy-resistant colorectal carcinoma clinically, Ricarda Herr et al. established a CDX mouse model based on human colorectal cancer cell lines whose B-Raf^V600E^ expression can be conditionally knocked down by doxycycline treatment, through which they revealed a novel facet of clinically applied B-Raf or MEK inhibitors by promoting cellular adhesion and differentiation of colorectal carcinoma cells (26). While long-term *in vitro* culture does result in the loss of many of the inherent features and heterogeneous characteristics of their parental cancer tissues. These shortcomings are most apparent when these cell lines are compared with their parental cancer strains from sick patients, implying that using these cell lines may compromise the value of any anticancer drug efficacy predictions in a clinical setting (27, 28). PDX mouse models are usually generated using mice with combined T/B/NK cell deficiency and macrophage tolerance for human cells, like NOD/SCID and NSG/NOG mice, which are repopulated with primary human cancer cells or tumor samples *in vivo*. Compared with CDX mouse models, PDX mouse models retain much more of their parental malignancy characteristics and are considered a more powerful tool for evaluating the effect of anticancer drugs in pre-clinical studies (29) (Table 2). For instance, Dr. Sidransky's group performed PDX studies in a large heterogeneous population (237 patients with various tumor types) and verified that human tumor grafts in PDX models can faithfully conserve genetic patterns of primary tumor. Additionally, their analysis further demonstrates that PDXs accurately replicate patients' clinical outcomes after treatments, indicating the capacity of this platform for assessment of anticancer drug efficacy (47).

**Table 2:** Advantages and applications of PDX mouse models for cancer study.

**Cancer**	**Advantages**	**Applications**	**References**
Lung cancer	Retain genetic and histological characteristics.	Predict the possibility of relapse after curative surgery.	(30)
Colorectal cancer	Retain the intratumor clonal heterogeneity and chromosomal instability.	Predict responsiveness to cetuximab in patients.	(31, 32)
Pancreatic cancer	Maintain the original tumor architecture; retain a greater proportion of stromal components and develop locoregional and distant metastases.	Demonstrate the activity of mitomycin C and cisplatin in a patient harboring a *PALB2* mutation. Demonstrate that stromal modulation may increase intra-tumor gemcitabine concentrations to improve therapy efficacy.	(33–35)
Head and neck cancer	Highly reflect promoter methylation in tumors and reproduced tumor heterogeneity.	Predict phase II clinical drug activity of cisplatin, diaziquone, pazelliptine, and retelliptine.	(36)
Breast cancer	Retain basal-like morphology and tumor structure.	Demonstrate the activity of cisplatin and ifosfamide combinatory therapy; evaluate the efficacy of trastuzumab.	(37, 38)
Glioblastoma multiforme	Retained genetic characteristics.	Assess the efficacy of bevacizumab.	(39, 40)
Renal cell carcinoma	Maintain the ability to evaluate tumor angiogenesis; Retain genetic and histological characteristics.	Evaluate the effects of sorafenib or sunitinib.	(41–43)
Prostate cancer	Exhibit the differentiation and expression of androgen receptor and prostate-specific antigen (PSA).	Predict the efficacy of androgen ablation therapy.	(44, 45)
Melanoma	Retain histology, genetic profiles, and tumor antigen characteristics.	Treatment with temozolomide exhibits similar responses to the corresponding patients.	(46)

### Mouse Models of Spontaneous Human Oncogenesis

One major shortcoming of both the CDX or PDX models for human oncogenesis is the lack of an oncologic transformation process from normal cells into malignant cells. Transplantation of healthy human cells in which tumor-suppressive genes were disrupted and/or oncogenes were overexpressed into immunodeficient mice has been used to simulate the entire oncogenesis process for a number of cancers, including leukemia, lymphoma, and melanoma (1) and so on (Table 3). For example, mice with spontaneous human acute human B lymphoblastic leukemia (B-ALL) were created by transplanting human CD34^+^ hematopoietic stem cells (HSCs) after transduction with retroviral vectors carrying *MLL-AF9* genes into immunodeficient mice, allowing the researchers to evaluate the underlying mechanisms of human B-ALL development (52). Similarly, seeding human melanocytes transformed with mutated melanoma-associated genes, including *N-Ras*^G12V^, *CDK4*^R24C^, and dominant-negative *p53*^R248W^, which are critical for p16^INK4A^-CDK4-Rb and ARF-HDM2-p53 tumor suppressor pathways, into autologous human skin grafts in immunodeficient mice results in the development of human melanocytic neoplasia *in vivo*, demonstrating the value of mouse models in the functional analysis and validation of mutations observed in human melanoma (53).

**Table 3:** Spontaneous human cancer model.

**Cancer**	**Method**	**References**
Myeloid neoplasia	Using CRISPR/Cas9 technology to introduce *FLT3-ITD* and *SMC3* mutation in CD34^+^ cells, and transplant them to NSG mice.	(48)
Lung cancer	Incorporate the mutated genes (*CDK4, hTERT, sh-p53, KRASV12*, and *c-MYC*) by lentiviral vectors into human bronchial epithelial cells, and transplant them to NOD/SCID mice.	(49)
T-ALL	Incorporate *NOTCH1*ΔE (N) and *LMO2/TAL1/BMI1* (LTB) gene by lentiviral vectors to into CD34^+^ cells, and transplant them to NSG mice.	(50)
AML	Incorporate *BCR-ABL* gene into CD34^+^ umbilical blood cells by retroviral vectors, and transplant them to NSG mice.	(51)
B-ALL and AML	Incorporate *MLL-AF* gene into CD34^+^ cells by retroviral vectors, and transplant them to BRG mice.	(52)
Melanoma	Incorporate *N-Ras*^G12V^, *CDK4*^R24C^, and dominant-negative *p53*^R248W^ into human melanocyte by retro-viral vectors, and transplant into the autologous human skin graft in CB.17 scid mice.	(53)

### Non-immune Factors Affect Human Oncogenesis in Mouse Models

Other than immunological factors, non-immune factors related to tumor-associated micro-environments may also influence the feasibility and quality of using mouse models to study human oncogenesis, especially for some human hematological malignancies (54). Aberrant gene expression in different stages of human HSC differentiation leads to a variety of hematological malignancies, including B cell leukemia, myeloid leukemia, and myeloma (55). The lack of or suboptimal interaction with mouse cytokines/chemokines/ligands, which are crucial for human hematological differentiation or cell survival may impair human oncogenesis in recipient mice (54). For example, poor interaction between mouse GM-CSF/IL-3 and IL-6 and human cells reduces the practicality of using immunodeficient mice to recapitulate human myeloid leukemia (56) and myeloma (57), respectively. The generation of immunodeficient mice with relevant human cytokine expression markedly improves their value in investigating aberrant human hematological complications, including acute myeloid leukemia (AML) (56), myeloma (57), chronic myelomonocytic leukemia (CMML) (58), juvenile myelomonocytic leukemia (JMML) (58), and myelodysplastic syndromes (MDS) (59). In addition, “humanization” of mouse bone marrow micro-environments by adding human stromal cells also facilitates the pathogenesis of human hematological disorders. For instance, Dr. Daniel Nowak's group showed that intra-bone injection of MDS patient-derived mesenchymal stromal cells (MSCs) contributes to the propagation of MDS-initiating stem cells and disease progression in orthotopic xenografts of NSG and NSG-SGM3 animals (NSG animals constitutively expresses human GM-CSF/IL-3 and stem cell factors) (60). Majeti et al. reported that an artificial human bone marrow (BM) microenvironment can be constructed by the subcutaneous injection of human BM-derived MSCs (humanized ossicles), which enables robust engraftment of healthy human HSCs as well as primary human leukemia-initiating cells from AML, acute promyelocytic leukemia (APL), and myelofibrosis (MF) (61) (Table 4).

**Table 4:** Improvement of mouse models for human hematological malignancy study.

**Strategy**	**Cancer**	**Model construction**	**References**
Human cytokine expression	MDS	Newborn MISTRG mice were intrahepatically injected with split-donor MDS BM CD34^+^ cells.	(59)
	AML	Newborn MISTRG mice were intrahepatically injected with primary favorable-risk AML cells.	(62)
	CMML	NSG-SGM3 mice were intravenously injected with CD34^+^ cells sorted from CMML patients' bone marrow or peripheral blood.	(58, 63)
	JMML	NSG-SGM3 mice were intravenously injected with CD34^+^ cells sorted from JMML patients' bone marrow or peripheral blood.	(58)
Human MSCs implantation	AML	Inject primary human AML cells into human BM-MSC formed ossicle in NSG mice.	(61)
	APL	Inject primary human APL cells into human BM-MSC formed ossicle in NSG mice.	(61)
	MDS	Human CD34^+^ cells and MSCs collected from MDS patients were simultaneously intra-bone injected into NSG mice.	(60)

## Humanized Mouse Models With Human Immune Systems in Cancer Research

It is widely accepted that immune surveillance is closely involved in oncogenesis and has a significant impact on treatment efficacies and outcomes (7). Additionally, the successful application of cancer therapeutic regimens, including co-stimulation signal blockades and adoptive transfers of anticancer immune subsets in treating metastatic malignancies, which show poor prognosis using traditional therapies, further highlights the importance of human immunity in the investigation of human oncology (8, 64). Therefore, humanized mice reconstructed with human immune systems are expected to aid the comprehensive study of the interactions between human cancer and human immunological elements.

Humanized mice with human immune systems have been extensively studied over the past three decades and have been reviewed in detail in several previous reports (10–12, 54). Various humanized mouse models, including the Hu-PBL (peripheral blood leukocyte)-SCID model (65), SRC (SCID repopulating cell)-Hu model (17, 66), and Thy/HSC (23, 67) [also named as BLT (68)] model, are commonly used in human oncology studies with each model having their own unique advantages and disadvantages (Table 5).

**Table 5:** Comparison between different HIS humanized mouse models.

	**Hu-PBL-SCID**	**SRC-Hu**	**Thy/HSC (BLT)**
Accessibility of human sample	Good	Moderate	Difficult (potential ethic problem)
Technique for model construction	Easy	Moderate	Relative difficult (required anesthesia and transplant technique)
Human immune cell survival/development	Majority of activated human T cells; Transient human B cells, myeloid cells and NK cells;	Multi-lineage human immune cell reconstitution; Poor human thymopoiesis; Lack of HLA mediate thymic selection for human T cells.	Multi-lineage human immune cell reconstitution; Robust human thymopoiesis; Human TCR repertoire influenced by mouse antigen.
Human immune function	T cell responses; Lack of interaction between human T cells, B cells, and myeloid cells.	Poor HLA restricted T cell responses; Poor T cell-dependent humoral responses.	Good HLA restricted T cell responses; Good T cell-dependent humoral response.
Time window	Short	Long	Long

### Hu-PBL-SCID Model

The Hu-PBL-SCID model is created by injecting human PBLs into immunodeficient mice, which transiently host multi-lineage human immune subsets (65). However, due to the lack of self-renewing human hematopoietic stem/progenitor cells and the relatively short life span of mature immune subsets, limited numbers of human myeloid cells and B cells engrafted in these mice. Instead, engrafted human immune cells primarily belong to activated human CD4^+^ or CD8^+^ T cell driven by mouse major histocompatibility (MHC) molecules, which cause severe xenogeneic graft vs. host diseases (xeno-GVHD), thereby restricting the experimental window for these animals to few weeks (69). Due to the relatively simple handling and accessibility of human PBL samples, the Hu-PBL-SCID model is widely used to study interactions between human immune cells, including T cells and NK cells, and human tumors *in vivo*. For example, Jakobsen et al. reported the feasibility of using a Hu-PBL-SCID model to evaluate the efficacy of a Bi-specific TCR (T cell receptor)-anti-CD3 regimen for treating NY-ESO-1- and LAGE-1-positive human tumors (70); Ignacio Melero et al. showed that this model can be used to study the effects of human PD-1 (Nivolumab) and CD137 (Urelumab) antibodies on the T cell-mediated anti-tumor response *in vivo* (71). Interestingly, Ryuzo Ueda et al. reported that Hu-PBL-SCID can also be used to study the anti-tumor effects driven by human NK cells through antibody-dependent cellular cytotoxic (ADCC) approaches (72). To overcome the constraints of xeno-GVHD, researchers went on to develop MHC knockout immunodeficient mice, like NOG-dKO or NOG-β2 m, IAβdKO mice, which demonstrate a reduced susceptibility to xeno-GVHD and extended experimental time frames (73, 74).

### SRC-Hu Model

The SRC-Hu model with human immunity is usually constructed by transplanting human hematopoietic stem/progenitor cells into neonatal or adult Il2 rg knockout immunodeficient recipients, like NOG (17, 75), BRG (66), NSG (76), or other similar murine strains. Here, mice present with a sustainable human immune system composed of human T cells, B cells, and myeloid cells. Due to the accessibility of cord blood and the relatively easy construction procedure, the SRC-Hu mouse model is one of the most popular humanized mouse models for the research of human immune relevant subjects (11). The SRC-Hu model is a valuable tool to be used to evaluate co-stimulatory molecule blockade effects and study anticancer drug effects in a physiologically relevant immune environment (77). For instance, Wang et al. showed that PD-1-targeted immunotherapy can be modeled in SRC-Hu humanized NSG mice, but not control NSG mice, bearing CDX and PDX partial HLA matched human tumor (referred to human immune system), demonstrating the value of the SRC-Hu model for cancer immunotherapy investigation (78). However, human T cells are educated in the mouse thymus and these animals have very poor human thymopoiesis (75). In addition, SRC-Hu mice cannot efficiently generate HLA-restricting antigen-specific immune responses (79). These limitations may restrict the value of the SRC-Hu model in human immune-oncology studies.

### Thy/HSC Model

In 2004, based on the Dr. McCune's Thy/Liv SCID-Hu mouse model (80), our group developed a novel humanized mouse model by co-transplantation of human fetal liver and thymic tissues into the renal capsule and intravenous injection of CD34^+^ human fetal liver cells (FLCs) into NOD/SCID mice after total body sublethal irradiation (67). These humanized mice have high levels of multilineage human lymphohematopoietic cell reconstitution, including human T cells, B cells, conventional dendritic cells (cDCs), plasmacytoid dendritic cells (pDCs), and macrophages, which can be detected in the blood as well as the lymphoid organs (23). Later, we found that human fetal liver tissue implantation is dispensable for human immune system reconstruction in this humanized mouse model, and named it as the Thy/HSC model (12). This method of construction was confirmed by Dr. J. Victor Garcia and colleagues in 2006 who named them BLT mice (68). Unlike the SRC-Hu model, robust human thymopoiesis occurs in engrafted human thymic tissues, in which typical thymic structures including cortex, medulla, and Hassall's bodies are formed by human stromal cells and human T cell progenitor cells can be identified (23). Importantly, Thy/HSC humanized mice can generate potent human immune responses as evidenced by the capability of these animals to reject allogeneic (81) and xenogeneic grafts (82), their generation of HLA-restricted antigen-specific human T cell reactions, and their ability to produce antigen-specific human IgM and IgG antibodies with subclass switching after immunization or xenograft implantation (82, 83). In Thy/HSC mice, mouse dendritic cells can migrate into human thymic grafts and may play a role in human T progenitor cell thymic education, thus explaining the reduced incidence of xeno-GVHD syndrome in Thy/HSC mice. Moreover, cryopreservation and “pipetting” of human thymic grafts before transplantation can eliminate existing human T cell progenitor cells, further alleviating xeno-GVHD syndrome, with some animals only experiencing xeno-GVHD more than 25 weeks post-transplant (84–86). For these reasons, Thy/HSC models are considered one of the most powerful *in vivo* tools for investigating human immune responses and their effects on therapeutic interventions (87) and are already widely applied in many biomedical fields including pathogen infection (88), allo/xeno-transplantation (81, 82), autoimmune diseases (89), regenerative medicine (90), immune molecule-targeting drug tests, and cancer research (91). For example, Thy/HSC mice can be modified to act as TCR transgenic humanized mice to study human T cell adaptive immunotherapy (92). Briefly, a melanoma antigen (MART-1)-specific TCR transgenic humanized mouse model was constructed by co-transplanting HLA-A^*^0201^+^ human fetal thymic tissues and autologous human CD34^+^ FLCs transduced with lentiviral vectors containing HLA-A^*^0201 restricted MART-1 specific TCR genes into sub-lethal irradiation pre-conditioned NSG mice, in which most MART-1-specific T cells contained only MART-1 TCR alpha and beta chains due to the allele exclusion process in the human thymic grafts (93). Using this tool, we revealed that the simultaneous inclusion of rapamycin for MART-1 TCR^+^ human T cell expansion *in vitro* and supplementation with human IL-15 *in vivo* greatly improves the anti-melanoma effects mediated by adoptive transfer of human MART-1 TCR^+^ T cells (92).

One of the main drawbacks of the Thy/HSC model is the requirement of human fetal samples for model construction, which are difficult to obtain by researchers due to ethnic problem and/or local policy (94). In addition, the TCR repertoire of human T cells generated in Thy/HSC mice may be different from the ones in human, because of the involvement of mouse antigen in human thymic educating process in Thy/HSC humanized mice.

## Improving Human Immunity in Humanized Mice For Cancer Research

Despite the fact that high human lymphohematopoietic chimerism can be constructed in Thy/HSC and SRC-Hu mice, there are still several limitations in the combination and functionality of the human immune system in humanized mice when compared with humans, which are primarily caused by the “unfriendly” mouse microenvironment for human immune cells to survival/differentiate/migrate/function (54). For example, there are significantly lower levels of myeloid cells and NK cells in humanized mice compared to human due to a lack of sufficient cross-talk between the mouse cytokines (GM-CSF/IL-3, M-CSF, Flt-3-l, IL-15, etc.) and the human cells, which limits their application in the study of the interactions between human myeloid/NK cells and human tumor tissues (54). Human immunity in humanized mice can be improved by supplementing the corresponding human cytokines by injecting recombinant proteins (95) or hydrodynamic injections of plasmids containing human cytokine genes (96) or using immunodeficient transgenic mice expressing the relevant proteins (97). Continuous overexpression of human cytokine (such as NSG-SGM3) may lead to abnormal human cell function; thus a series of human cytokine knock-in (human gene driven by mouse promotor) immunodeficient mouse strains (such as MISTRG) were developed to ensure an appropriate tissue-, cell-, and context-specific expression of the incorporated protein (98–100). These strategies greatly expand the application of these humanized mice in immune and cancer research. For example, Dr. Richard Flavell's group showed that humanized mice made using MISTRG (human M-CSF, GM-CSF/IL-3, TPO knock in Rag2^−/−^Il2rg^−/−^ mice with transgenic human *Sirpa* expression) mice showed significantly improved cellular ratios as well as function of human myeloid and NK cells in blood and lymphoid organs when compared to Rag2^−/−^ Il2rg^−/−^ or NSG mice (100). In addition, they also showed that human macrophage infiltration in a human tumor xenograft in MISTRG humanized mice used a similar mechanism to those exhibited in human tumor biopsy samples (100). In another report, they showed that humanized SRG-15 mice (knock-in with human IL-15 and *Sirpa* gene in Rag2^−/−^Il2rg^−/−^ mice) have dramatically improved NK cell development and function. These cells demonstrate similar expression patterns of killer inhibitory receptors when compared to NK cells from human donors, which is an important breakthrough in the application of humanized mice in the study of human NK cell-mediated and combinatory cancer immunotherapy strategies *in vivo* (20).

Although development of human cytokine transgenic/knock-in immunodeficient mice optimizes human immune function, researchers must still be aware of the potential interference resulting from the persistent secretion of these inflammatory human cytokines in recipient mice. Our recent studies have shown that Thy/HSC humanized mice made on NSG-SGM3 but not NSG mice showed dramatically reduced life spans and increased incidence of hemophagocytic lymphohistiocytosis (HLH) syndrome, as evidenced by elevated levels of human inflammatory cytokines, including IL-6, IL-4, IL-10, IFN-γ, TNF-α, IL-18, severe anemia/thrombocytopenia phenomena, and aberrant activation and infiltration of human macrophages and T cells in systemic organs (85). Similar phenomena were also observed in SRC-Hu models made using NSG-SGM3 (101) mice and MISTRG mice (102). Bondanza et al. reported that CAR-T cells induced cytokine-release syndrome (CRS) and neurotoxicity caused by the secretion of IL-1 and IL-6 by human monocytes can be modeled in humanized mice made using NSG-SGM3 mice but not in NSG mice, which also highlights the important role of inflammatory cytokine secretion in animal mortality and aberrant human immune activation (103). Thus, interpretation of the results collected from humanized mice made using immunodeficient recipients with abnormal human cytokine secretion may need to consider these factors.

## Humanized Mouse Models of Human Oncogenesis Under Autologous Human Immune Surveillance

**Figure 1:**
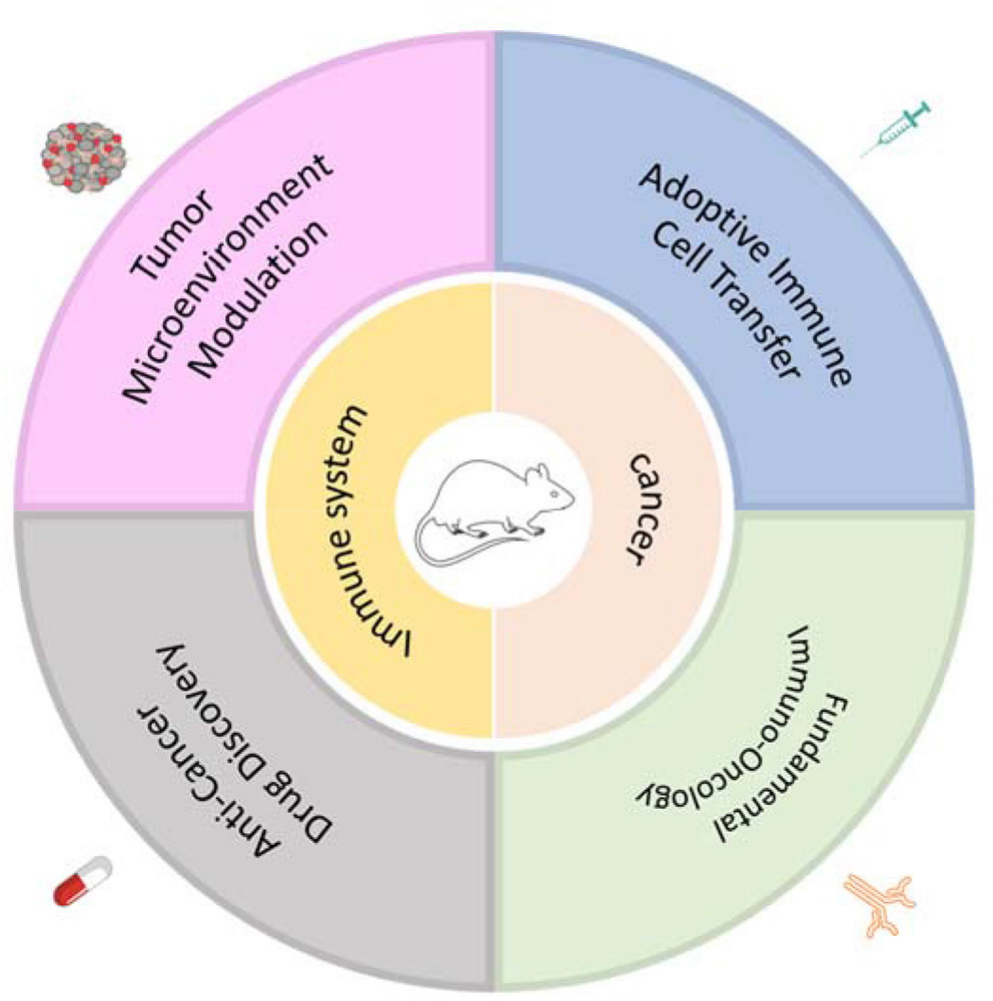
Application of humanized mouse models with human oncogenesis under autologous human immune surveillance for cancer research.

One major limitation of most humanized mouse models created for human cancer study is that these reconstructed human immune systems are allogeneic to the inoculated human tumors (102). Thus, robust allogeneic responses conferred by human T cells to human tumors may compromise the value of the data collected from these models, making it difficult to extend the observations from these humanized mice to the complex interactions between the tumor antigen-specific T cells and human tumor tissues in patients, or precisely predicting anticancer drug effects (102). As such, the development of humanized mouse models in which human oncogenesis occurs under autologous functional human immune surveillance is critical to the field's continued development (Figure 1).

Recently, we developed a humanized mouse model with spontaneous development of human B-ALL under autologous human immune surveillance by incorporating the leukemia-associated fusion gene, *MLL-AF9*, into human CD34^+^ FLCs, which were then co-transplanted with human fetal thymus tissue into NSG mice (104). Human B-ALL collected from these humanized mice can survive and expand into the Thy/HSC humanized mice made by autologous but not allogeneic human fetal samples. Using this model, we were able to show that recipient leukocyte infusions (RLI), a GVHD-free immunotherapeutic approach, markedly reduced human leukemia burden during induced lymphopenia, further validating the safety and efficacy of human RLI for human leukemia treatment (104). Moreover, this model can also be utilized to study chimeric antigen T (CAR-T) cell therapy, which still requires modification to improve anticancer function while restricting various side effects, including CRS and neurotoxicity. We showed that Thy/HSC mice with autologous B-ALL treated with anti-CD19 CAR-T cells exhibited similar kinetics and levels to those observed in patients, and rapid production of T cell- and myeloid cell-derived cytokines, such as GM-CSF, IFN-γ, TNF-α, and IL-10, and elevation of regulatory T cell frequency, which has been reported in patients receiving CAR-T therapy, were also found in this mouse model (105). These results indicate that these animal models could be reliably used to characterize human CAR-T cell function *in vivo* and facilitate the development of novel CAR therapies.

In addition, a novel humanized mouse model, named PDXv2.0, was recently constructed by Dr. Jonas A. Nilsson's group through adoptive transfer of *in vitro* expanded human tumor infiltrating lymphocytes (TILs) into the PDX mice that host the tumor collected from the same patients (106). They showed that the PDX2.0 mouse model made by immunodeficient mouse recipients with human IL-2 continuous production efficiently represents the reactivity of adoptive cell transfer (ACT) immunotherapy that occurred in patients, offering a powerful platform to model ACT-based immunotherapies as well as combinatory therapies for heterogeneous human cancers (106). Based on a similar model, Dr. Ignacio Melero et al. verified that transient expression of IL-12 mRNA in human antitumor CD8^+^ T cells by electroporation can markedly improve their antitumor effects after intratumor adoptive transfer (107).

## Discussion and Concluding Remarks

When compared to traditional rodent models, humanized rodents provide a much closer approximation of human physiology and pathology with broader application in basic cancer research and anticancer drug/approach discovery. While more effective methods of construction, design, and functionality for these models will still be required to make them able to address specific concerns and boost translation of basic cancer research. We hypothesize that the optimization of humanized mice in cancer research may result from the following scenarios: (1) Invention of humanized mouse models with human immune systems and autologous human oncogenesis for more types of human tumors including melanoma, lung cancer, hepatocarcinoma, and colorectal cancer, in which pluripotent stem cell technology (108) and gene editing tools, such as CRISPR/Cas9, may play crucial roles (109); (2) Development of humanized mouse models with functional human adaptive and innate immunity that do not require human fetal or even cord blood samples, to reduce ethical considerations and broaden their application (94, 110); for example, substitution of human fetal thymic tissue with HLA transgenic porcine thymic tissue (111) to make Thy/HSC models or the generation of humanized mice using pluripotent stem cell-derived human hematopoietic stem/progenitor cells (112) and human thymic epithelial cells (113); (3) Development of immunodeficient mouse strains with relevant human cytokine/chemokine/ligand secretion under physiological conditions to promote reconstructed human immunity without aberrant human immune disorder; (4) Establishment of humanized large animal models, such as humanized pig models, in which human cancer therapy could be modeled at more physiologically relevant scales and closer physiological conditions using more relevant timelines (114); (5) Generation of personalized humanized mouse models (86) that can simultaneously host primary human cancer samples and reconstitute the patients' unique immune system as a personalized platform to substitute patients for anticancer therapy tests.

In summary, humanized mouse models with optimized designs could offer a more powerful tool to not only better understand the roles of human immune elements in human cancer development and treatment but also facilitate the invention and translation of novel anticancer therapeutic drugs/approaches in the future.

## Author Contributions

HT and YL performed literature search and wrote the manuscript. Y-GY and ZH conceived the framework of this article, wrote, and edited the manuscript. All authors contributed to the article and approved the submitted version.

## Conflict of Interest

The authors declare that the research was conducted in the absence of any commercial or financial relationships that could be construed as a potential conflict of interest.

## References

[1] Zitvogel L Pitt JM Daillere R Smyth MJ Kroemer G. Mouse models in oncoimmunology. Nat Rev Cancer. (2016) 16:759–73. 10.1038/nrc.2016.91 27687979

[2] Landgraf M McGovern JA Friedl P Hutmacher DW. Rational design of mouse models for cancer research. Trends Biotechnol. (2018) 36:242–51. 10.1016/j.tibtech.2017.12.001 29310843

[3] Perrin S. Preclinical research: make mouse studies work. Nature. (2014) 507:423–5. 10.1038/507423a 24678540

[4] Fogh J Fogh JM Orfeo T. One hundred and twenty-seven cultured human tumor cell lines producing tumors in nude mice. J Natl Cancer Inst. (1977) 59:221–6. 10.1093/jnci/59.1.221 327080

[5] Hidalgo M Amant F Biankin AV Budinska E Byrne AT Caldas C. Patient-derived xenograft models: an emerging platform for translational cancer research. Cancer Discov. (2014) 4:998–1013. 10.1158/2159-8290.CD-14-0001 25185190 PMC4167608

[6] Aparicio S Hidalgo M Kung AL. Examining the utility of patient-derived xenograft mouse models. Nat Rev Cancer. (2015) 15:311–6. 10.1038/nrc3944 25907221

[7] Chen DS Mellman I. Elements of cancer immunity and the cancer-immune set point. Nature. (2017) 541:321–30. 10.1038/nature21349 28102259

[8] Wolchok JD Kluger H Callahan MK Postow MA Rizvi NA Lesokhin AM. Nivolumab plus ipilimumab in advanced melanoma. N Engl J Med. (2013) 369:122–33. 10.1056/NEJMoa1302369 23724867 PMC5698004

[9] Grupp SA Kalos M Barrett D Aplenc R Porter DL Rheingold SR. Chimeric antigen receptor-modified T cells for acute lymphoid leukemia. N Engl J Med. (2013) 368:1509–18. 10.1056/NEJMoa1215134 23527958 PMC4058440

[10] Shultz LD Ishikawa F Greiner DL. Humanized mice in translational biomedical research. Nat Rev Immunol. (2007) 7:118–30. 10.1038/nri2017 17259968

[11] Shultz LD Brehm MA Garcia-Martinez JV Greiner DL. Humanized mice for immune system investigation: progress, promise and challenges. Nat Rev Immunol. (2012) 12:786–98. 10.1038/nri3311 23059428 PMC3749872

[12] Hu Z Yang YG. Human lymphohematopoietic reconstitution and immune function in immunodeficient mice receiving cotransplantation of human thymic tissue and CD34(+) cells. Cell Mol Immunol. (2012) 9:232–6. 10.1038/cmi.2011.63 22307039 PMC3346882

[13] Yang YG Sykes M. Xenotransplantation: current status and a perspective on the future. Nat Rev Immunol. (2007) 7:519–31. 10.1038/nri2099 17571072

[14] Mombaerts P Iacomini J Johnson RS Herrup K Tonegawa S Papaioannou VE. RAG-1-deficient mice have no mature B and T lymphocytes. Cell. (1992) 68:869–77. 10.1016/0092-8674(92)90030-G 1547488

[15] Shinkai Y Rathbun G Lam KP Oltz EM Stewart V Mendelsohn M. RAG-2-deficient mice lack mature lymphocytes owing to inability to initiate V(D)J rearrangement. Cell. (1992) 68:855–67. 10.1016/0092-8674(92)90029-C 1547487

[16] Bosma GC Custer RP Bosma MJ. A severe combined immunodeficiency mutation in the mouse. Nature. (1983) 301:527–30. 10.1038/301527a0 6823332

[17] Ito M Hiramatsu H Kobayashi K Suzue K Kawahata M Hioki K. NOD/SCID/gamma(c)(null) mouse: an excellent recipient mouse model for engraftment of human cells. Blood. (2002) 100:3175–82. 10.1182/blood-2001-12-0207 12384415

[18] Christianson SW Greiner DL Hesselton RA Leif JH Wagar EJ Schweitzer IB. Enhanced human CD4+ T cell engraftment in beta2-microglobulin-deficient NOD-scid mice. J Immunol. (1997) 158:3578–86. 9103418

[19] Takenaka K Prasolava TK Wang JC Mortin-Toth SM Khalouei S Gan OI. Polymorphism in sirpa modulates engraftment of human hematopoietic stem cells. Nat Immunol. (2007) 8:1313–23. 10.1038/ni1527 17982459

[20] Herndler-Brandstetter D Shan L Yao Y Stecher C Plajer V Lietzenmayer M. Humanized mouse model supports development, function, and tissue residency of human natural killer cells. Proc Natl Acad Sci USA. (2017) 114:E9626–34. 10.1073/pnas.1705301114 29078283 PMC5692533

[21] Yamauchi T Takenaka K Urata S Shima T Kikushige Y Tokuyama T. Polymorphic Sirpa is the genetic determinant for NOD-based mouse lines to achieve efficient human cell engraftment. Blood. (2013) 121:1316–25. 10.1182/blood-2012-06-440354 23293079

[22] Bendle GM Linnemann C Hooijkaas AI Bies L de Witte MA Jorritsma A. Schumacher: lethal graft-versus-host disease in mouse models of T cell receptor gene therapy. Nat Med. (2010) 16:565–70. 10.1038/nm.2128 20400962

[23] Lan P Tonomura N Shimizu A Wang S Yang YG. Reconstitution of a functional human immune system in immunodeficient mice through combined human fetal thymus/liver and CD34+ cell transplantation. Blood. (2006) 108:487–92. 10.1182/blood-2005-11-4388 16410443

[24] Shultz D Lyons BL Burzenski LM Gott B Chen X Chaleff S. Human lymphoid and myeloid cell development in NOD/LtSz-scid IL2R gamma null mice engrafted with mobilized human hemopoietic stem cells. J Immunol. (2005) 174:6477–89. 10.4049/jimmunol.174.10.6477 15879151

[25] Nakamura M Suemizu H. Novel metastasis models of human cancer in NOG mice. Curr Top Microbiol Immunol. (2008) 324:167–77. 10.1007/978-3-540-75647-7_11 18481460

[26] Herr R Kohler M Andrlova H Weinberg F Moller Y Halbach S. B-Raf inhibitors induce epithelial differentiation in BRAF-mutant colorectal cancer cells. Cancer Res. (2015) 75:216–29. 10.1158/0008-5472.CAN-13-3686 25381152

[27] Gillet JP Calcagno AM Varma S Marino M Green LJ Vora MI. Redefining the relevance of established cancer cell lines to the study of mechanisms of clinical anti-cancer drug resistance. Proc Natl Acad Sci USA. (2011) 108:18708–13. 10.1073/pnas.1111840108 22068913 PMC3219108

[28] Hausser HJ Brenner RE. Phenotypic instability of Saos-2 cells in long-term culture. Biochem Biophys Res Commun. (2005) 333:216–22. 10.1016/j.bbrc.2005.05.097 15939397

[29] Gao H Korn JM Ferretti S Monahan JE Wang Y Singh M. High-throughput screening using patient-derived tumor xenografts to predict clinical trial drug response. Nat Med. (2015) 21:1318–25. 10.1038/nm.3954 26479923

[30] Fichtner I Rolff J Soong R Hoffmann J Hammer S Sommer A. Establishment of patient-derived non-small cell lung cancer xenografts as models for the identification of predictive biomarkers. Clin Cancer Res. (2008) 14:6456–68. 10.1158/1078-0432.CCR-08-0138 18927285

[31] Guenot D Guerin E Aguillon-Romain S Pencreach E Schneider A Neuville A. Primary tumour genetic alterations and intra-tumoral heterogeneity are maintained in xenografts of human colon cancers showing chromosome instability. J Pathol. (2006) 208:643–52. 10.1002/path.1936 16450341

[32] Fichtner I Slisow W Gill J Becker M Elbe B Hillebrand T. Anticancer drug response and expression of molecular markers in early-passage xenotransplanted colon carcinomas. Eur J Cancer. (2004) 40:298–307. 10.1016/j.ejca.2003.10.011 14728946

[33] Olive P Jacobetz MA Davidson CJ Gopinathan A McIntyre D Honess D. Inhibition of hedgehog signaling enhances delivery of chemotherapy in a mouse model of pancreatic cancer. Science. (2009) 324:1457–61. 10.1126/science.1171362 19460966 PMC2998180

[34] Villarroel C Rajeshkumar NV Garrido-Laguna I De Jesus-Acosta A Jones S Maitra A. Personalizing cancer treatment in the age of global genomic analyses: PALB2 gene mutations and the response to DNA damaging agents in pancreatic cancer. Mol Cancer Ther. (2011) 10:3–8. 10.1158/1535-7163.MCT-10-0893 21135251 PMC3307340

[35] Von Hoff DD Ramanathan RK Borad MJ Laheru DA Smith LS Wood TE. Gemcitabine plus nab-paclitaxel is an active regimen in patients with advanced pancreatic cancer: a phase I/II trial. J Clin Oncol. (2011) 29:4548–54. 10.1200/JCO.2011.36.5742 21969517 PMC3565012

[36] Langdon SP Hendriks HR Braakhuis BJ Pratesi G Berger DP Fodstad O. Preclinical phase II studies in human tumor xenografts: a European multicenter follow-up study. Ann Oncol. (1994) 5:415–22. 10.1093/oxfordjournals.annonc.a058872 8075048

[37] de Plater L Lauge A Guyader C Poupon MF F de Cremoux AP Vincent-Salomon A. Establishment and characterisation of a new breast cancer xenograft obtained from a woman carrying a germline BRCA2 mutation. Br J Cancer. (2010) 103:1192–200. 10.1038/sj.bjc.6605900 20877358 PMC2967069

[38] DeRose YS Wang G Lin YC Bernard PS Buys SS Ebbert MT. Tumor grafts derived from women with breast cancer authentically reflect tumor pathology, growth, metastasis and disease outcomes. Nat Med. (2011) 17:1514–20. 10.1038/nm.2454 22019887 PMC3553601

[39] Keunen O Johansson M Oudin A Sanzey M Rahim SA Fack F. Anti-VEGF treatment reduces blood supply and increases tumor cell invasion in glioblastoma. Proc Natl Acad Sci USA. (2011) 108:3749–54. 10.1073/pnas.1014480108 21321221 PMC3048093

[40] Wang J Miletic H Sakariassen PO Huszthy PC Jacobsen H Brekka N. A reproducible brain tumour model established from human glioblastoma biopsies. BMC Cancer. (2009) 9:465. 10.1186/1471-2407-9-465 20040089 PMC2810304

[41] Grisanzio C Seeley A Chang M Collins M Di Napoli A Cheng SC. Orthotopic xenografts of RCC retain histological, immunophenotypic and genetic features of tumours in patients. J Pathol. (2011) 225:212–21. 10.1002/path.2929 21710693 PMC3793840

[42] Yuen S Sim MY Siml HG Chong TW Lau WK Cheng CW. Inhibition of angiogenic and non-angiogenic targets by sorafenib in renal cell carcinoma (RCC) in a RCC xenograft model. Br J Cancer. (2011) 104:941–7. 10.1038/bjc.2011.55 21407223 PMC3065286

[43] Hammers HJ Verheul HM Salumbides B Sharma R Rudek M Jaspers J. Reversible epithelial to mesenchymal transition and acquired resistance to sunitinib in patients with renal cell carcinoma: evidence from a xenograft study. Mol Cancer Ther. (2010) 9:1525–35. 10.1158/1538-7445.AM10-388 20501804 PMC3049816

[44] Yoshida T Kinoshita H Segawa T Nakamura E Inoue T Shimizu Y. Antiandrogen bicalutamide promotes tumor growth in a novel androgen-dependent prostate cancer xenograft model derived from a bicalutamide-treated patient. Cancer Res. (2005) 65:9611–6. 10.1158/0008-5472.CAN-05-0817 16266977

[45] Wang Y Revelo MP Sudilovsky D Cao M Chen WG Goetz L. Development and characterization of efficient xenograft models for benign and malignant human prostate tissue. Prostate. (2005) 64:149–59. 10.1002/pros.20225 15678503

[46] Nemati F Sastre-Garau X Laurent C Couturier J Mariani P Desjardins L Establishment and characterization of a panel of human uveal melanoma xenografts derived from primary and/or metastatic tumors. Clin Cancer Res. (2010) 16:2352–62. 10.1158/1078-0432.CCR-09-3066 20371695

[47] Izumchenko E Paz K Ciznadija D Sloma I Katz A Vasquez-Dunddel D. Patient-derived xenografts effectively capture responses to oncology therapy in a heterogeneous cohort of patients with solid tumors. Ann Oncol. (2017) 28:2595–605. 10.1093/annonc/mdx416 28945830 PMC5834154

[48] Tothova Z Krill-Burger JM Popova KD Landers CC Sievers QL Yudovich D. Multiplex CRISPR/Cas9-based genome editing in human hematopoietic stem cells models clonal hematopoiesis and myeloid neoplasia. Cell Stem Cell. (2017) 21:547–55 e8. 10.1016/j.stem.2017.07.015 28985529 PMC5679060

[49] Sato M Larsen JE Lee W Sun H Shames DS Dalvi MP. Human lung epithelial cells progressed to malignancy through specific oncogenic manipulations. Mol Cancer Res. (2013) 11:638–50. 10.1158/1541-7786.MCR-12-0634-T 23449933 PMC3687022

[50] Kusakabe M Sun AC Tyshchenko K Wong R Nanda A Shanna C. Synthetic modeling reveals HOXB genes are critical for the initiation and maintenance of human leukemia. Nat Commun. (2019) 10:2913. 10.1038/s41467-019-10510-8 31266935 PMC6606637

[51] Sontakke P Carretta M Jaques J Brouwers-Vos AZ Lubbers-Aalders L Yuan H. Modeling BCR-ABL and MLL-AF9 leukemia in a human bone marrow-like scaffold-based xenograft model. Leukemia. (2016) 30:2064–73. 10.1038/leu.2016.108 27125308

[52] Barabe F Kennedy JA Hope KJ Dick JE. Modeling the initiation and progression of human acute leukemia in mice. Science. (2007) 316:600–4. 10.1126/science.1139851 17463288

[53] Chudnovsky Y Adams AE Robbins PB Lin Q Khavari PA. Use of human tissue to assess the oncogenic activity of melanoma-associated mutations. Nat Genet. (2005) 37:745–9. 10.1038/ng1586 15951821 PMC3063773

[54] Rongvaux A Takizawa H Strowig T Willinger T Eynon EE Flavell RA. Human hemato-lymphoid system mice: current use and future potential for medicine. Annu Rev Immunol. (2013) 31:635–74. 10.1146/annurev-immunol-032712-095921 23330956 PMC4120191

[55] Weigert O Weinstock DM. The evolving contribution of hematopoietic progenitor cells to lymphomagenesis. Blood. (2012) 120:2553–61. 10.1182/blood-2012-05-414995 22869790

[56] Feuring-Buske M Gerhard B Cashman J Humphries RK Eaves CJ E D. Hogge: Improved engraftment of human acute myeloid leukemia progenitor cells in beta 2-microglobulin-deficient NOD/SCID mice and in NOD/SCID mice transgenic for human growth factors. Leukemia. (2003) 17:760–3. 10.1038/sj.leu.2402882 12682634

[57] Das R Strowig T Verma R Koduru S Hafemann A Hopf S. Microenvironment-dependent growth of preneoplastic and malignant plasma cells in humanized mice. Nat Med. (2016) 22:1351–7. 10.1038/nm.4202 27723723 PMC5101153

[58] Yoshimi A Balasis ME Vedder A Feldman K Ma Y Zhang H. Robust patient-derived xenografts of MDS/MPN overlap syndromes capture the unique characteristics of CMML and JMML. Blood. (2017) 130:397–407. 10.1182/blood-2017-01-763219 28576879 PMC5533204

[59] Song Y Rongvaux A Taylor A Jiang T Tebaldi T Balasubramanian K. A highly efficient and faithful MDS patient-derived xenotransplantation model for pre-clinical studies. Nat Commun. (2019) 10:366. 10.1038/s41467-018-08166-x 30664659 PMC6341122

[60] Medyouf H Mossner M Jann JC Nolte F Raffel S Herrmann C. Myelodysplastic cells in patients reprogram mesenchymal stromal cells to establish a transplantable stem cell niche disease unit. Cell Stem Cell. (2014) 14:824–37. 10.1016/j.stem.2014.02.014 24704494

[61] Reinisch A Thomas D Corces MR Zhang X Gratzinger D Hong WJ. A humanized bone marrow ossicle xenotransplantation model enables improved engraftment of healthy and leukemic human hematopoietic cells. Nat Med. (2016) 22:812–21. 10.1038/nm.4103 27213817 PMC5549556

[62] Ellegast JM Rauch PJ Kovtonyuk LV Muller R Wagner U Saito Y. inv(16) and NPM1mut AMLs engraft human cytokine knock-in mice. Blood. (2016) 128:2130–4. 10.1182/blood-2015-12-689356 27581357 PMC5084606

[63] Hudson E de Wolski K Kapp LM Richards AL Schniederjan MJ Zimring JC. Antibodies to senescent antigen and C3 are not required for normal red blood cell lifespan in a murine model. Front Immunol. (2017) 8:1425. 10.3389/fimmu.2017.01425 29163500 PMC5670101

[64] Rizvi NA Hellmann MD Snyder A Kvistborg P Makarov V Havel JJ. Chan: Cancer immunology. Mutational landscape determines sensitivity to PD-1 blockade in non-small cell lung cancer. Science. (2015) 348:124–8. 10.1126/science.aaa1348 25765070 PMC4993154

[65] Mosier DE Gulizia RJ Baird SM Wilson DB. Transfer of a functional human immune system to mice with severe combined immunodeficiency. Nature. (1988) 335:256–9. 10.1038/335256a0 2970594

[66] Traggiai E Chicha L Mazzucchelli L Bronz L Piffaretti JC Lanzavecchia A. Development of a human adaptive immune system in cord blood cell-transplanted mice. Science. (2004) 304:104–7. 10.1126/science.1093933 15064419

[67] Lan P Wang L Diouf B Eguchi H Su H Bronson R. Induction of human T-cell tolerance to porcine xenoantigens through mixed hematopoietic chimerism. Blood. (2004) 103:3964–9. 10.1182/blood-2003-10-3697 14739221

[68] Melkus MW Estes JD Padgett-Thomas A Gatlin J Denton PW Othieno FA. Humanized mice mount specific adaptive and innate immune responses to EBV and TSST-1. Nat Med. (2006) 12:1316–22. 10.1038/nm1431 17057712

[69] Tary-Lehmann M Lehmann PV Schols D Roncarolo MG Saxon A. Anti-SCID mouse reactivity shapes the human CD4+ T cell repertoire in hu-PBL-SCID chimeras. J Exp Med. (1994) 180:1817–27. 10.1084/jem.180.5.1817 7964463 PMC2191753

[70] McCormack E Adams KJ Hassan NJ Kotian A Lissin NM Sami M. Bi-specific TCR-anti CD3 redirected T-cell targeting of NY-ESO-1- and LAGE-1-positive tumors. Cancer Immunol Immunother. (2013) 62:773–85. 10.1007/s00262-012-1384-4 23263452 PMC3624013

[71] Sanmamed MF Rodriguez I Schalper KA Onate C Azpilikueta A Rodriguez-Ruiz ME. Melero: nivolumab and urelumab enhance antitumor activity of human T lymphocytes engrafted in rag2-/-il2rgammanull immunodeficient mice. Cancer Res. (2015) 75:3466–78. 10.1158/0008-5472.CAN-14-3510 26113085

[72] Ito A Ishida T Yano H Inagaki A Suzuki S Sato F. Defucosylated anti-CCR4 monoclonal antibody exercises potent ADCC-mediated antitumor effect in the novel tumor-bearing humanized NOD/Shi-scid, IL-2Rgamma(null) mouse model. Cancer Immunol Immunother. (2009) 58:1195–206. 10.1007/s00262-008-0632-0 19048251 PMC11030985

[73] Ashizawa T Iizuka A Nonomura C Kondou R Maeda C Miyata H. Antitumor effect of programmed death-1 (PD-1) blockade in humanized the NOG-MHC double knockout mouse. Clin Cancer Res. (2017) 23:149–58. 10.1158/1078-0432.CCR-16-0122 27458246

[74] Yaguchi T Kobayashi A Inozume T Morii K Nagumo H Nishio H. Human PBMC-transferred murine MHC class I/II-deficient NOG mice enable long-term evaluation of human immune responses. Cell Mol Immunol. (2018) 15:953–62. 10.1038/cmi.2017.106 29151581 PMC6207709

[75] Ishikawa F Yasukawa M Lyons B Yoshida S Miyamoto T Yoshimoto G. Development of functional human blood and immune systems in NOD/SCID/IL2 receptor {gamma} chain(null) mice. Blood. (2005) 106:1565–73. 10.1182/blood-2005-02-0516 15920010 PMC1895228

[76] Kumar P Ban HS Kim SS Wu H Pearson T Greiner DL. T cell-specific siRNA delivery suppresses HIV-1 infection in humanized mice. Cell. (2008) 134:577–86. 10.1016/j.cell.2008.06.034 18691745 PMC2943428

[77] Zhao Y Shuen TWH Toh TB Chan XY Liu M Tan SY. Development of a new patient-derived xenograft humanised mouse model to study human-specific tumour microenvironment and immunotherapy. Gut. (2018) 67:1845–54. 10.1136/gutjnl-2017-315201 29602780 PMC6145285

[78] Wang M Yao LC Cheng M Cai D Martinek J Pan CX. Humanized mice in studying efficacy and mechanisms of PD-1-targeted cancer immunotherapy. FASEB J. (2017) 32:1537–49. 10.1096/fj.201700740R 29146734 PMC5892726

[79] Shultz LD Saito Y Najima Y Tanaka S Ochi T Tomizawa M. Generation of functional human T-cell subsets with HLA-restricted immune responses in HLA class I expressing NOD/SCID/IL2r gamma(null) humanized mice. Proc Natl Acad Sci USA. (2010) 107:13022–7. 10.1073/pnas.1000475107 20615947 PMC2919921

[80] McCune JM Namikawa R Kaneshima H Shultz LD Lieberman M Weissman LI. The SCID-hu mouse: murine model for the analysis of human hematolymphoid differentiation and function. Science. (1988) 241:1632–9. 10.1126/science.2971269 2971269

[81] Rong Z Wang M Hu Z Stradner M Zhu S Kong H. An effective approach to prevent immune rejection of human ESC-derived allografts. Cell Stem Cell. (2014) 14:121–30. 10.1016/j.stem.2013.11.014 24388175 PMC4023958

[82] Tonomura N Shimizu A Wang S Yamada K Tchipashvili V Weir GC. Pig islet xenograft rejection in a mouse model with an established human immune system. Xenotransplantation. (2008) 15:129–35. 10.1111/j.1399-3089.2008.00450.x 18447886

[83] Tonomura N Habiro K Shimizu A Sykes M Yang YG. Antigen-specific human T-cell responses and T cell-dependent production of human antibodies in a humanized mouse model. Blood. (2008) 111:4293–6. 10.1182/blood-2007-11-121319 18270327 PMC2288728

[84] Tang Y Yang YG Bai O Xia J Hu Z. Long-term survival and differentiation of human thymocytes in human thymus-grafted immunodeficient mice. Immunotherapy. (2019) 11:881–8. 10.2217/imt-2019-0030 31140331 PMC6949514

[85] Yoshihara S Li Y Xia J Danzl N Sykes M Yang YG. Posttransplant hemophagocytic lymphohistiocytosis driven by myeloid cytokines and vicious cycles of T-cell and macrophage activation in humanized mice. Front Immunol. (2019) 10:186. 10.3389/fimmu.2019.00186 30814997 PMC6381030

[86] Kalscheuer H Danzl N Onoe T Faust T Winchester R Goland R. A model for personalized *in vivo* analysis of human immune responsiveness. Sci Translat Med. (2012) 4:125ra30. 10.1126/scitranslmed.3003481 22422991 PMC3697150

[87] Manz MG Di Santo PJ. Renaissance for mouse models of human hematopoiesis and immunobiology. Nat Immunol. (2009) 10:1039–42. 10.1038/ni1009-1039 19767720

[88] Brainard DM Seung E Frahm N Cariappa A Bailey CC Hart WK. Induction of robust cellular and humoral virus-specific adaptive immune responses in human immunodeficiency virus-infected humanized BLT mice. J Virol. (2009) 83:7305–21. 10.1128/JVI.02207-08 19420076 PMC2704767

[89] Tan S Li Y Xia J Jin CH Hu Z Duinkerken G. Type 1 diabetes induction in humanized mice. Proc Natl Acad Sci USA. (2017) 114:10954–9. 10.1073/pnas.1710415114 28874533 PMC5642714

[90] Zhao T Zhang ZN Westenskow PD Todorova D Hu Z Lin T. Humanized mice reveal differential immunogenicity of cells derived from autologous induced pluripotent stem cells. Cell Stem Cell. (2015) 17:353–9. 10.1016/j.stem.2015.07.021 26299572 PMC9721102

[91] Walsh NC Kenney LL Jangalwe S Aryee KE Greiner DL Brehm MA. Humanized mouse models of clinical disease. Annu Rev Pathol. (2017) 12:187–215. 10.1146/annurev-pathol-052016-100332 27959627 PMC5280554

[92] Hu Z Xia J Fan W Wargo J Yang YG. Human melanoma immunotherapy using tumor antigen-specific T cells generated in humanized mice. Oncotarget. (2016) 7:6448–59. 10.18632/oncotarget.7044 26824989 PMC4872726

[93] Li Y Teteloshvili N Tan S Rao S Han A Yang YG. Humanized mice reveal new insights into the thymic selection of human autoreactive CD8(+) T cells. Front Immunol. (2019) 10:63. 10.3389/fimmu.2019.00063 30778347 PMC6369192

[94] Reardon S. Trump administration halts fetal-tissue research by government scientists. Nature. (2019) 570:148. 10.1038/d41586-019-01783-6 31186554

[95] Huntington ND Legrand N Alves NL Jaron B Weijer K Plet A. IL-15 trans-presentation promotes human NK cell development and differentiation *in vivo*. J Exp Med. (2009) 206:25–34. 10.1084/jem.20082013 19103877 PMC2626663

[96] Chen Q Khoury M Chen J. Expression of human cytokines dramatically improves reconstitution of specific human-blood lineage cells in humanized mice. Proc Natl Acad Sci USA. (2009) 106:21783–8. 10.1073/pnas.0912274106 19966223 PMC2789167

[97] Billerbeck E Barry WT Mu K Dorner M Rice CM Ploss A. Development of human CD4+FoxP3+ regulatory T cells in human stem cell factor-, granulocyte-macrophage colony-stimulating factor-, and interleukin-3-expressing NOD-SCID IL2Rgamma(null) humanized mice. Blood. (2011) 117:3076–86. 10.1182/blood-2010-08-301507 21252091 PMC3062310

[98] Rathinam C Poueymirou WT Rojas J Murphy AJ Valenzuela DM Yancopoulos GD. Efficient differentiation and function of human macrophages in humanized CSF-1 mice. Blood. (2011) 118:3119–28. 10.1182/blood-2010-12-326926 21791433

[99] Willinger T Rongvaux A Takizawa H Yancopoulos GD Valenzuela DM Murphy AJ. Human IL-3/GM-CSF knock-in mice support human alveolar macrophage development and human immune responses in the lung. Proc Natl Acad Sci USA. (2011) 108:2390–5. 10.1073/pnas.1019682108 21262803 PMC3038773

[100] Rongvaux A Willinger T Martinek J Strowig T Gearty SV Teichmann LL Development and function of human innate immune cells in a humanized mouse model. Nat Biotechnol. (2014) 32:364–72. 10.1038/nbt.2858 24633240 PMC4017589

[101] Wunderlich M Stockman C Devarajan M Ravishankar N Sexton C Kumar AR. A xenograft model of macrophage activation syndrome amenable to anti-CD33 and anti-IL-6R treatment. JCI Insight. (2016) 1:e88181. 10.1172/jci.insight.88181 27699249 PMC5033750

[102] De La Rochere P Guil-Luna S Decaudin D Azar G Sidhu SS Piaggio E. Humanized mice for the study of immuno-oncology. Trends Immunol. (2018) 39:748–63. 10.1016/j.it.2018.07.001 30077656

[103] Norelli M Camisa B Barbiera G Falcone L Purevdorj A Genua M. Monocyte-derived IL-1 and IL-6 are differentially required for cytokine-release syndrome and neurotoxicity due to CAR T cells. Nat Med. (2018) 24:739–48. 10.1038/s41591-018-0036-4 29808007

[104] Xia J Hu Z Yoshihara S Li Y Jin CH Tan S. Modeling human leukemia immunotherapy in humanized mice. EBioMedicine. (2016) 10:101–8. 10.1016/j.ebiom.2016.06.028 27394641 PMC5006579

[105] Jin CH Xia J Rafiq S Huang X Hu Z Zhou X. Modeling anti-CD19 CAR T cell therapy in humanized mice with human immunity and autologous leukemia. EBioMedicine. (2019) 39:173–81. 10.1016/j.ebiom.2018.12.013 30579863 PMC6354733

[106] Jespersen H Lindberg MF Donia M Soderberg EMV Andersen R Keller U. Clinical responses to adoptive T-cell transfer can be modeled in an autologous immune-humanized mouse model. Nat Commun. (2017) 8:707. 10.1038/s41467-017-00786-z 28955032 PMC5617838

[107] Etxeberria I Bolanos E Quetglas JI Gros A Villanueva A Palomero J. Intratumor adoptive transfer of IL-12 mRNA transiently engineered antitumor CD8(+) T cells. Cancer Cell. (2019) 36:613–29 e7. 10.1016/j.ccell.2019.10.006 31761658

[108] Rowe RG Daley GQ. Induced pluripotent stem cells in disease modelling and drug discovery. Nat Rev Genet. (2019) 20:377–88. 10.1038/s41576-019-0100-z 30737492 PMC6584039

[109] Ran FA Hsu PD Lin CY Gootenberg JS Konermann S Trevino AE. Double nicking by RNA-guided CRISPR Cas9 for enhanced genome editing specificity. Cell. (2013) 154:1380–9. 10.1016/j.cell.2013.08.021 23992846 PMC3856256

[110] Fantasy politics over fetal-tissue research Nature. (2017) 541:133 10.1038/541133a. Available online at: https://www.nature.com/news/fantasy-politics-over-fetal-tissue-research-1.21263 28079097

[111] Habiro K Sykes M Yang YG. Induction of human T-cell tolerance to pig xenoantigens via thymus transplantation in mice with an established human immune system. Am J Transplant. (2009) 9:1324–9. 10.1111/j.1600-6143.2009.02646.x 19459808 PMC2752337

[112] Sugimura R Jha DK Han A Soria-Valles C da Rocha EL Lu YF. Haematopoietic stem and progenitor cells from human pluripotent stem cells. Nature. (2017) 545:432–38. 10.1038/nature22370 28514439 PMC5872146

[113] Sun X Xu J Lu H Liu W Miao Z Sui X. Directed differentiation of human embryonic stem cells into thymic epithelial progenitor-like cells reconstitutes the thymic microenvironment *in vivo*. Cell Stem Cell. (2013) 13:230–6. 10.1016/j.stem.2013.06.014 23910085

[114] Boettcher N Loving CL Cunnick JE Tuggle CK. Development of severe combined immunodeficient (SCID) pig models for translational cancer modeling: future insights on how humanized SCID pigs can improve preclinical cancer research. Front Oncol. (2018) 8:559. 10.3389/fonc.2018.00559 30560086 PMC6284365

